# Assessment of Cytokine Expression Profile in Acute Myeloid Leukemia Patients Before and After Chemotherapy

**DOI:** 10.4274/tjh.2012.0164

**Published:** 2014-06-10

**Authors:** Zargham Sepehrizadeh, Mohammad Mohammadi, Amirhossein Emami, Mojtaba Tabatabaei Yazdi, Saeed Hashemi Bozchlou, Mohammad Reza Khorramizadeh, Mina Bahrololoumi Shapourabadi, Elham Jaberi, Naghmeh Rajaei, Neda Setayesh

**Affiliations:** 1 Tehran University of Medical Sciences Faculty of Pharmacy, Department of Pharmaceutical Biotechnology, Tehran, Iran; 2 Tehran University of Medical Sciences, Biotechnology Research Center, Tehran, Iran; 3 Tehran University of Medical Sciences Fa culty of Medicine, Department of Hematology and Oncology, Tehran, Iran; 4 Tehran University of Medical Sciences Faculty of Public Health, Department of Pathobiology, Tehran, Iran

**Keywords:** Acute myeloid leukemia, cytokines, Granulocyte colony-stimulating factor, RT-PCR

## Abstract

**Objective:** One of the major goals of cancer treatment is the monitoring of chemotherapeutic protocols. Quantitative and comparative cytokine expression profiling could be reliable to be used for biomarkers in deadly and fast-growing cancers such as acute myeloid leukemia (AML). The present study aims to assess and further validate cytokines with probable effects on proliferation and maturation of blood cells in AML.

**Materials and Methods:** Gene expression levels of IL-1β, IL-10, IL-8, TNF-α, and IFN-γ were analyzed before and after chemotherapy and after granulocyte colony-stimulating factor (G-CSF) therapy in 46 AML patients by an in-house quantitative comparative RT-PCR method.

**Results:** Our findings indicated that although the gene expression level of TNF-α was almost constant in all 3 samples, IL-1β, IL-8, and IL-10 expression levels showed a decrease after chemotherapy and an increase after G-CSF therapy. On the other hand, the expression level of IFN-γ had a different pattern with an increase after chemotherapy and a decrease after G-CSF therapy.

**Conclusion:** Taken together, the results of this study are in support of the idea that the analyzed cytokines could be useful biomarkers for AML treatment monitoring. However, further molecular epidemiological investigations are suggested to elaborate more cancer monitoring biomarkers.

## Abstract (tr)

**Amaç:** Kanser tedavisinin en önemli hedeflerinden bir tanesi de kemoterapi protokollerinin monitörizasyonudur. Niceliksel ve karşılaştırılabilir sitokin ekspresyon profili, akut myeloid lösemi (AML) gibi hızlı ilerleyen ve ölümcül kanserlerde biyobelirteç olarak kullanılmaya uygun görünmektedir. Bu çalışmada, sitokinlerin AML’deki kan hücrelerinin çoğalma ve olgunlaşması üzerine olası etkilerinin değerlendirilmesi ve daha da ötesi doğrulanması amaçlanmıştır.

**Gereç ve Yöntemler:** Hastanede yatan 46 AML hastasında, kemoterapi öncesi, sonrası ve granülosit koloni sitimüle edici faktör (G-CSF) uygulaması sonrası olacak şekilde, niceliksel karşılaştırmalı RT-PCR yöntemi ile IL-1β, IL-10, IL-8, TNF-α ve IFN-γ’nin gen ekspresyon düzeyleri analiz edildi.

**Bulgular:** Sonuçlarımız, TNF-α gen ekspresyon düzeyinin her üç durumda da sabit kalmasına karşın, IL-1β, IL-8 ve IL-10 ekspresyon düzeylerinin kemoterapi uygulamasından sonra düşerken, G-CSF tedavisinin ardından ise yükseldiğini ortaya koydu. Diğer yandan; IFN-γ ekspresyon düzeyleri ise, kemoterapiden sonra yükselip, G-CSF tedavisinin ardından düşerek tamamen farklı bir paterne sahip olduğunu gösterdi.

**Sonuç:** Birlikte ele alındığında, AML tedavisinin monitörizasyonunda sitokin ekspresyon analizinin yararlı bir biyobelirteç olduğu düşüncesi bu çalışma sonuçları ile destek bulmuştur. Bununla birlikte, gelecek epidemiyolojik moleküler çalışmaların çok daha fazla kanser takip biyobelirtecinin belirlenmesinde yol gösterici olacağı aşikardır.

## INTRODUCTION

Acute myeloid leukemia (AML) is a heterogeneous group of diseases with respect to biology and clinical course. It is characterized by the rapid proliferation of abnormal cells that accumulate in the bone marrow and interfere with the production of normal blood cells. The majority of patients are above 60 years of age, and they usually receive less intensive chemotherapy due to a high risk of treatment-related mortality [1,2]. Mechanisms enabling malignant cells in AML to escape physiologic growth restrictions are largely unknown. The presence of some cytokines can significantly affect the primary result of cytokine action in the processes of diseases [3,4,5]. The cytokines are soluble proteins that form a network that plays a key role in the immunoregulation of lymphocyte function [6,7]. A number of reports suggest that the growth of AML cells is controlled by cytokines produced by the leukemic cells in an autocrine or paracrine way. Unlike normal hematopoietic cells, leukemic blasts from many patients with AML constitutively express cytokines like IL-1, GM-CSF, G-CSF, IL-6, IL-8, TNF-α, and SCF [8,9,10,11].

IL-1β is a proinflammatory cytokine with a broad spectrum of local and systematic activities. It stimulates T helper cells and promotes the proliferation of B cells and lymphocytes. In the case of leukemic blast cells, it is suggested that the proliferation and generation of myeloid progenitor cells is an indirect effect of IL-1β via increasing of the colony-stimulating factor [12,13,14,15]. TNF-α is another proinflammatory cytokine with a wide spectrum of biological activities from the induction of cytological aspects to modulation of the production of other cytokines. In the progenitor lymphocytes, TNF-α stimulates the production of colony-stimulating factors. IFN-γ is an immunomodulator and cell proliferation inhibitor factor. An abnormal level of TNF-α in a patient with AML is probably attributed to the pathogenesis of AML [16]. It can affect AML cell proliferation both when cells are cultured alone and in the presence of nonleukemic stromal cells [17]. IL-8 is an antiinflammatory factor that acts as a chemoattractant for neutrophils in inflammation. IL-10 is an inhibitor of the production of proinflammatory cytokines. Is has been postulated that cytokines are working in a broad network; they modulate the expression of each other and have different and complex biological aspects. 

Granulocyte colony-stimulating factor (G-CSF) stimulates the proliferation and differentiation of neutrophils and granulocytes. Clinically, G-CSF is used for recovery and the increasing of the neutrophil counts of patients after chemotherapy courses to overcome febrile neutropenia [18].

The expression of cytokines could be considered as a criterion for evaluation of disease and treatment. The expression of some cytokines has been studied in vitro or in vivo in the literature and, surprisingly, there are different and even controversial reports in this field. In this study, we evaluated the expression of IL-1β, IL-10, IL-8, TNF-α, and IFN-γ cytokines in Iranian AML patients before and after chemotherapy and after G-CSF to validate the biomarker monitoring role of these cytokines in AML. 

## MATERIALS AND METHODS

**Sample Collection**

Blood samples were obtained from 46 consenting patients with AML at 3 intervals: before and after chemotherapy and after G-CSF therapy. Control samples were obtained from normal, healthy people of the same age and sex. All procedures were approved by an ethics committee and the consent of the participants was obtained.

**RNA Extraction**

For total RNA extraction, 3-mL blood samples were collected in sterile polypropylene tubes containing 4 mL of guanidine isothiocyanate (5.4 M) solution. The samples were mixed well and were subjected to total RNA extraction with the Roche High Pure RNA extraction kit according to the manufacturer’s instructions (Roche, Germany). The quality of extracted RNA was checked by denatured agarose gel electrophoresis and its quantity was calculated by biophotometer (Eppendorf, Germany) at 260 nm.

**Reverse Transcription Polymerase Chain Reaction **

Reverse transcription (RT) was performed using 1 µg of total RNA, 1 U of M-MuLV reverse transcriptase (Expand Reverse Transcriptase, Roche), 20 pmol of oligo(dT)18, 1 µL of dNTPs (10 mmol each), 2 µL of dithiothreitol, and 4 µL of reaction buffer (5X) in a total volume of 20 µL at 42 °C for 60 min.

Polymerase chain reaction (PCR; 25 cycles) was performed using 2 µL of synthesized cDNA, 2.5 U of SuperTaq DNA polymerase (ABgene, UK), 0.5 µL of dNTPs (10 mmol each), 2.5 µL of reaction buffer (10X), 1.5 mmol MgCl2, and 20 pmol of specific primers for each cytokine as represented in Table 1 in a total volume of 25 µL. 

**PCR Product Analysis**

PCR products were analyzed by 1% agarose gel electrophoresis. The density of resultant bands was calculated with UVIdoc software (Geldoc, UVItec, UK). The results for each sample were normalized by β-actin gene expression level and the relative gene expression was calculated. The calculated densitometric results for relative gene expression analyses were depicted as arbitrary units.

## RESULTS

Twenty-nine of 46 patients were female and 17 were male. The mean age of patients was 35 years. The minimum age of patients was 15 and the maximum age was 63. Thirteen of 46 patients were diagnosed with AML type m2 and 9 with m4, respectively (Tables 2, 3, 4).

**Figure 1:**
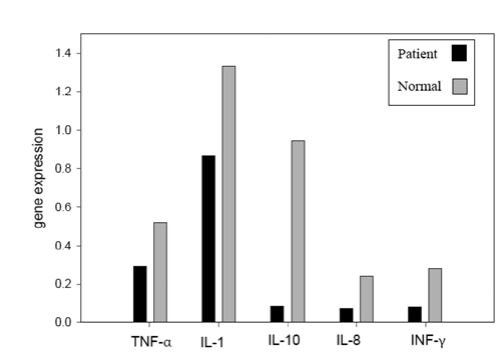
Gene expression levels of target genes in normal controls and patients.

Our findings showed that gene expression of cytokines such as TNF-α, IL-1β, IL-8, INF-γ, and IL-10 in AML patients were lower than in normal controls (Figure 1).

**Figure 2:**
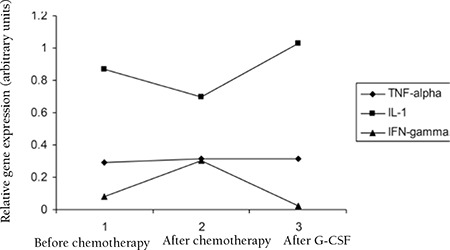
Expression level of TNF-α, IL-1β, and IFN-γ before and after chemotherapy and after G-CSF.

Mean gene expression for TNF-α before chemotherapy was 0.2931±0.061, after chemotherapy was 0.3140±0.070, and after G-CSF was 0.3148±0.0926; for normal controls it was 0.5195±0.07 (Figure 2).

Mean gene expression for IL-1β before chemotherapy was 0.8696±0.1570, after chemotherapy was 0.6978±0.1460, and after G-CSF was 1.0291±0.2528; for normal controls it was 1.3326±0.08 (Figure 2).

**Figure 3:**
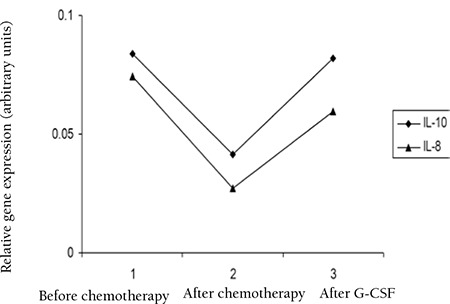
Expression level of IL-10 and IL-8 before and after chemotherapy and after G-CSF.

Mean gene expression for IFN-γ before chemotherapy was 0.0809±0.0235, after chemotherapy was 0.3032±0.2089, and after G-CSF was 0.0253±0.0168; for normal controls it was 0.2831±0.06 (Figure 3).

Mean gene expression for IL-10 before chemotherapy was 0.0836±0.0353, after chemotherapy was 0.0416±0.0182, and after G-CSF was 0.0817±0.0808; for normal controls it was 0.9435±0.07 (Figure 3).

Mean gene expression for IL-8 before chemotherapy was 0.0744±0.0256, after chemotherapy was 0.0272±0.0134, and after G-CSF was 0.0596±0.0384; for normal controls it was 0.2358±0.04 (Figure 3).

## DISCUSSION

There exist numerous studies in the literature that have investigated the gene expression profile of cytokines in patients suffering from AML and other diseases [19,20]. The most significant result of these studies is the great difference of gene expression profiles of leukemic cells in vivo and in vitro. In vitro studies overall show a high expression level of cytokines in cultured leukemic cells due to their maturation and cytokine production in culture medium, but in vivo studies report controversial results. The different in vivo results could stem from the different genotypes in patients or the complexity of the illness from the molecular point of view. For example, Reddy et al. reported a low level of expression for IFN-γ and TNF-α but a high level of expression of IL-8 in AML patients in comparison to healthy controls [21], but Gao et al. reported the high-level expression of TNF-α and IL-1β in AML patients [22]. Our findings showed that the mean gene expression levels of IL-1β (1.5-fold), TNF-α (1.8-fold), IL-8 (3-fold), IL-10 (11-fold), and IFN-γ (3.5-fold) in healthy normal controls were higher than in the AML patients at a steady state before chemotherapy. The low-level expression of cytokines in patients may be due to the depression of the immune system in AML patients and accumulation of immature cells in the blood, or it could be associated with abnormal Th1 to Th2 subpopulation shifting and, hence, activity. 

The results of this study showed that after chemotherapy, the gene expression level of TNF-α does not have any significant increase compared to before chemotherapy, but there is a significant increase in the expression level of IFN-γ after chemotherapy while the expression of IL-1β, IL-10, and IL-8 is decreased after chemotherapy as compared to the state before chemotherapy. The decrease of gene expression level of the mentioned cytokines is probably associated with the cytotoxic effect of chemotherapy on the blood cells, but this could not explain the increase of IFN-γ.

The gene expression levels of IL-1β, TNF-α, IL-8, and IL-10 were increased after G-CSF, which could be due to the effect of G-CSF in recovery of the immune system and the stimulation and release of cytokines. The increase of IL-8 and IL-10 reached the same level as before chemotherapy, but in the case of IL-1β, the level increased to higher than the levels before chemotherapy. The expression level of IFN-γ decreased after chemotherapy, probably due to the complex effect of other cytokines on IFN-γ production. 

Taken together, it could be concluded that the expression level of TNF-α in all 3 samples (before and after chemotherapy and after G-CSF) remains almost constant without any significant change. In the case of IL-1β, IL-8, and IL-10, gene expression decreased after chemotherapy and increased after G-CSF therapy, leading to consideration of these cytokines as potentially useful means for AML chemotherapy monitoring. IFN-γ shows a different pattern of gene expression, increased after chemotherapy and decreased after G-CSF treatment. 

Considering the complexity and plurality of cytokines’ gene expression profiles in cancer processes, our findings in AML patients show the promising monitoring biomarker potentials of some cytokines. However, further molecular epidemiological investigations on cytokine gene expression patterns in various therapeutic protocols are suggested to elaborate more cancer monitoring biomarkers.

## ACKNOWLEDGMENT

This study was financially sponsored by a research project registered by the Deputy of Research, Tehran University of Medical Sciences, Tehran, Iran. The authors would also like to acknowledge the technical assistance kindly offered by A. Araghi from Imam Khomeini Hospital, Tehran University of Medical Sciences, Tehran, Iran.

## CONFLICT OF INTEREST STATEMENT

The authors of this paper have no conflicts of interest, including specific financial interests, relationships, and/or affiliations relevant to the subject matter or materials included. 

## Figures and Tables

**Table 1:**
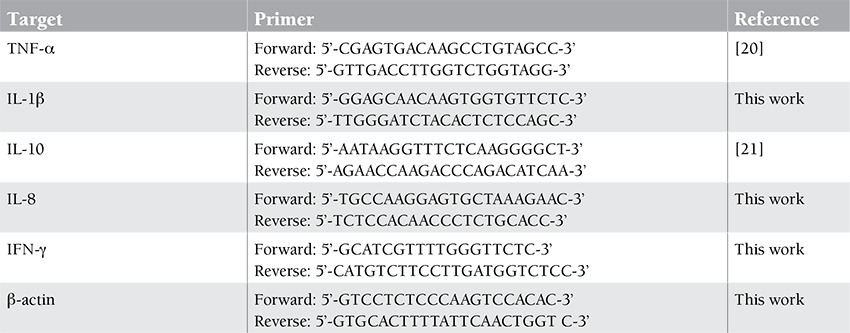
Patients’ chemotherapy outcome.

**Table 2:**
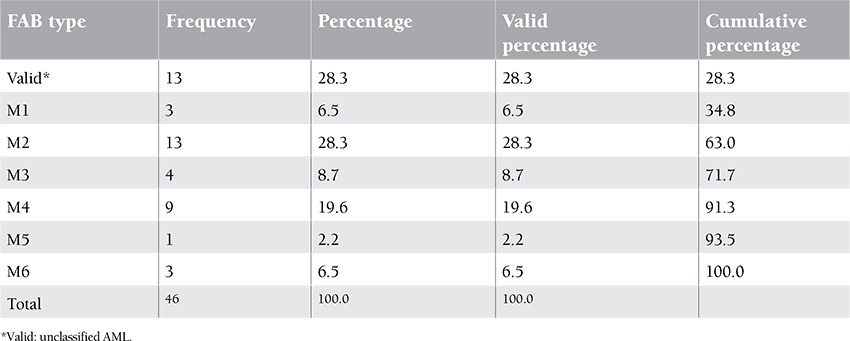
Patients’ standard clinical typing: type determination, frequency, and percentage of the studied patients.

**Table 3:**
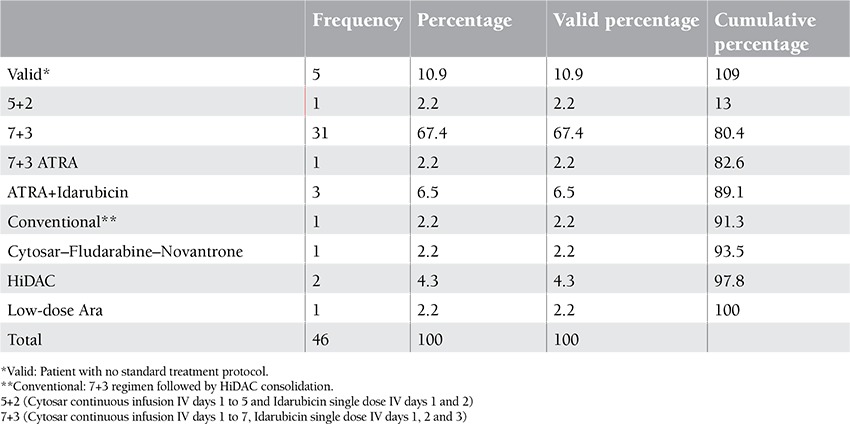
Patients’ chemotherapy protocols.

**Table 4:**
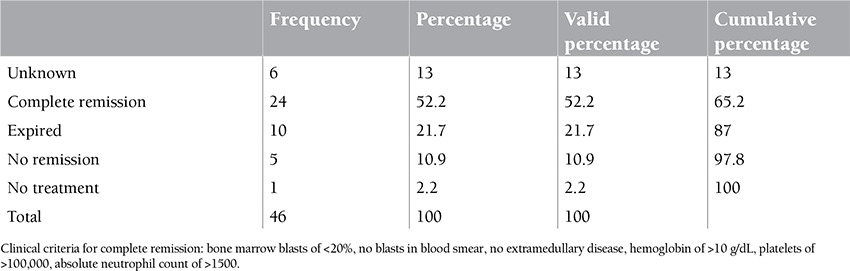
Patients’ chemotherapy outcome.
